# Neurological Implications of Non-critically Ill Patients With Coronavirus Disease 2019 in a Fangcang Shelter Hospital in Wuhan, China

**DOI:** 10.3389/fneur.2020.00895

**Published:** 2020-08-26

**Authors:** Nao Yan, Zhipeng Xu, Bin Mei, Yongzhe Gao, Dongwei Lv, Junjian Zhang

**Affiliations:** Department of Neurology, Zhongnan Hospital of Wuhan University, Wuhan, China

**Keywords:** neurologic symptoms, COVID-19, SARS-CoV-2, CNS, neurological implications

## Abstract

**Background:** Coronavirus disease 2019 (COVID-19) is a new viral respiratory disease and has become a pandemic. Fever, weakness, and dry cough are the main clinical manifestations. However, little is known about neurological symptoms of non-critically ill COVID-19 patients.

**Objective:** To investigate the neurological symptoms and implications of patients with non-critically ill COVID-19 patients.

**Materials and Methods:** This retrospective cohort study investigated all COVID-19 patients admitted to Wuhan East-West Lake Fangcang shelter hospital. Demographic data, clinical manifestations, comorbidities, radiological data, the result of nucleic acid test, and treatments were collected and analyzed.

**Results:** Among 1,682 patients with confirmed non-critically ill COVID-19, 509 patients (30.3%) had neurological symptoms, including myalgia (311, 18.5%), headache (216, 12.8%), fatigue (83, 4.9%), and dizziness (15, 0.9%). One hundred and fourteen patients (6.8%) were the expansion of pulmonary infection according to their chest CT images and medical history. Compared with patients without neurological symptoms, patients with neurological symptoms had a significantly longer length of hospital stay, time of nucleic acid turning negative, and the mean time from onset of symptom to hospital admission (*p* < 0.05). Patients with neurological symptoms were more likely to occur the expansion of pulmonary infection compared with the patients without neurological symptoms (46/509 [9.0%] vs. 68/1,173 [5.8%]).

**Conclusions:** Non-critically ill COVID-19 patients commonly have neurological symptoms. Neurological symptoms are significantly associated with the processes of COVID-19. Early identification and aggressive treatment are particularly important for COVID-19 patients with neurological symptoms.

## Introduction

COVID-19 is a potentially severe acute respiratory infection caused by severe acute respiratory syndrome coronavirus 2 (SARS-CoV-2), which has spread rapidly around the world (1–4). On March 11, the World Health Organization (WHO) declared that the COVID-19 outbreak can be characterized as a pandemic (5). As of 27 June 2020, the data from WHO indicate that there have been 9,653,048 confirmed cases, including 491,128 deaths in just 4 months, which has affected more than 200 countries and regions.

The human respiratory system is recognized as the SARS-CoV-2 primary target. Fever, cough, and dyspnea are the most common clinical symptoms at the onset of illness (6, 7). Moreover, several studies have confirmed that SARS-CoV-2 is highly similar to SARS-CoV; the latter can invade human multiple systems including lung, trachea/bronchus, stomach, small intestine, brain, and liver, not just the respiratory system (8–11). Recent researches have shown that digestive symptoms are common in patients with COVID-19, including nausea, vomiting, and diarrhea, and the fecal–oral route may be a potential route for SARS-CoV-2 infection (12–16). Some experts hold the view that SARS-CoV-2 may invade the brain and central nervous system by the ACE2 receptor (17, 18), which is present in the nervous system and skeletal muscles.

A recent study showed that 81% of SARS-CoV-2 nucleic acid-positive patients were classified as mild (non-critically ill patients), which displays non-pneumonia or only mild pneumonia (19). In order to address the drawbacks of home isolation and ease pressures on the designated traditional hospitals, 15 Fangcang shelter hospitals special for non-critically ill COVID-19 patients were rapidly established by the urgent renovation of stadiums and exhibition centers. The diagnostic and classification criteria of COVID-19 are based on the diagnosis and treatment program for novel coronavirus pneumonia issued by the National Health Commission (NHC) of the People's Republic of China (Trial Version 6). All patients who are nucleic acid positive, have mild to moderate symptoms (respiratory rate <30 breaths/min and blood oxygen saturation >93% at resting state), and are quarantined at home are unified transferred to nearby Fangcang shelter hospitals by the government (20–23).

At present, most of the studies focused on the clinical characteristics of severe or critically ill patients. However, specific information characterizing neurological symptoms of non-critically ill COVID-19 patients remains obscure. The present study aims to investigate the neurological implications of non-critically ill COVID-19 patients and provide references for the prevention and treatment of the disease.

## Materials and Methods

This single-center retrospective cohort study was performed at Wuhan East-West Lake Fangcang shelter hospital, which is the first largest shelter hospital designated by the government to admitting non-critically ill COVID-19 patients in the city, constructed and led by Zhongnan Hospital of Wuhan University from February 4, 2020, to March 10, 2020. We retrospectively analyzed all COVID-19 patients admitted to Wuhan East-West Lake Fangcang shelter hospital. Moreover, we only excluded a few patients with incomplete data. All patients were confirmed COVID-19 by real-time reverse transcriptase-polymerase chain reaction (RT-PCR) in designated hospitals before admission. Throat-swab specimens were obtained for the nucleic acid test.

The diagnostic and classification criteria issued by the National Health Commission (NHC) of the People's Republic of China (Trial Version 6) classified the disease severity of patients to 4 levels: mild, moderate, severe, and critical. The mild grade represents patients with mild clinical symptoms and no sign of pneumonia on imaging. The moderate grade represents patients who have a fever and respiratory symptoms with radiologic findings of pneumonia. The severe grade represents patients meet any of the following criteria: (1) respiratory distress (≥30 breaths/min); (2) oxygen saturation ≤ 93% at rest; and (3) PaO_2_FiO_2_ ≤ 300 mm Hg or chest imaging showing obvious lesion progression >50% within 24–48 h. The critical grade represents patients meeting any of the following criteria: (1) respiratory failure and requiring mechanical ventilation; (2) shock; (3) having other organ failures that require ICU care (24).

### Data Collection

A trained team of physicians reviewed electronic medical records, nursing records, laboratory findings, and radiologic examinations for all patients during the epidemic period. Patient data including demographic, medical history, chest CT imaging, treatments, the result of nucleic acid test, and comorbidities were collected and analyzed. Clinical symptoms from onset to hospital admission were provided by COVID-19 patients who were conscious, cognitively, and mentally normal.

### Statistical Analysis

Continuous variables were described as mean ± standard deviation (SD) or median (interquartile range, IQR) and compared with independent group *t*-test or the Mann–Whitney U test if appropriate. Categorical variables were described as a percentage and compared by χ^2^ test or Fisher's exact test if appropriate. All statistical analyses were performed using SPSS 22.0 software (SPSS Inc. Chicago, IL). A value of *P* < 0.05 at two-sided was considered statistically significant.

## Results

### Clinical Characteristics of Patients With Non-critically Ill COVID-19

A total of 1,682 patients with COVID-19 were included in the analysis, and 17 patients were excluded due to incomplete data. The demographic and clinical characteristics are shown in Table 1. The median [IQR] age was 50 [39–58] years (range, 5–89), and 859 patients (51.1%) were female. Of these patients, the median [IQR] day of hospital stay was 19 (14–23) days (range 1–29). The median [IQR] day of nucleic acid turning negative for all patients was 9 (5–13) days (range 0–25). The majority of these non-critically ill COVID-19 patients have no comorbidities. The most common comorbidities were hypertension (118, 7.0%), followed by diabetes (53, 3.2%); lung disease (36, 2.2%), such as chronic bronchitis (30, 1.8%), bronchiectasis (3, 0.2%), and tuberculosis (3, 0.2%); coronary heart disease (15, 0.9%); and cerebral infarction (5, 0.3%). After admission, antiviral and antibiotic basic medical care is provided. There were 1,617 patients (96.1%) receiving traditional Chinese medicine (Qingfei Paidutang, QPD) treatment, which is recommended by the National Health Commission (NHC) of the People's Republic of China; 1,372 (81.4%) on Lianhua Qingwen capsule; 1,355 (80.6%) on antivirals including umifenovir (1316, 78.2%), Lopinave/Litonawe (164, 9.8%), oseltamivir (62, 3.7%), and tenofovir (2,0.1%), and 899 (53.4%) on antibiotics including moxifloxacin hydrochloride (863,51.3%), cephalosporin (103, 6.1%), and azithromycin (12,7.1%), 2 (0.1%) on glucocorticoids (budesonide). Based on the results of chest CT images and medical history, 114 patients (6.8%) made up the expansion of pulmonary infection during hospitalization.

**Table 1:** Clinical characteristics of patients with non-critically ill COVID-19.

**Characteristics**	**No. (%)**
No. of patients	1,682
Age, median [IQR], (range), years	50 [39–58], (5–89)
<30	125 (7.4)
30–45	508 (30.2)
46–65	892 (53.0)
≥65	157 (9.3)
**Gender**	
Female	859 (51.1)
Male	823 (48.9)
From onset of symptom to hospital admission, median [IQR] (range), days	10 (7–15), (1–60)
Hospital stay, median [IQR] (range), days	19 (14–23), (1–29)
**Comorbidities**	
Hypertension	118 (7.0)
Diabetes	53 (3.2)
**Lung disease**	
Chronic bronchitis	30 (1.8)
Bronchiectasis	3 (0.2)
Tuberculosis	3 (0.2)
Coronary heart disease	15 (0.9)
Cerebral infarction	5 (0.3)
Turning to nucleic acid negative, median [IQR] (range), days	9 (5–13), (0–25)
**Treatments**	
Traditional Chinese Medicine (Qingfei Paidutang, QPD)	1,617 (96.1)
Lianhua Qingwen capsule	1,372 (81.4)
Antibiotics	899 (53.4)
Moxifloxacin hydrochloride	863 (51.3)
Cephalosporin	103 (6.1)
Azithromycin	12 (7.1)
Antivirals	1,355 (80.6)
Umifenovir	1,316 (78.2)
Lopinave/Litonawe (62, 3.7%)	164 (9.8)
Oseltamivir	62 (3.7)
Tenofovir	2 (0.1)
Glucocorticoid (budesonide)	2 (0.1)
Expansion of pulmonary infection	114 (6.8)

*IQR, interquartile range; COVID-19, Coronavirus disease 2019. The results were presented as median [IQR] (range) for continuous variables and number (%) for categorical variables*.

### Clinical Symptoms of Patients With Non-critically Ill COVID-19 on Admission

The median [IQR] day of the onset of symptoms to hospital admission for all patients was 10 (7–15) days (range 1–60). Clinical symptoms of all patients on admission are summarized in Table 2. Respiratory system symptoms are the most common clinical symptoms (1,559, 92.7%), including fever (1,177, 70.1%), cough (952, 56.6%), chest tightness or dyspnea (425, 25.3%), expectoration (363, 21.4%), chills (285, 16.9%), sore throat (254, 15.1%), nasal obstruction (113, 6.7%), and runny nose (107, 6.4%). The second prevalence of clinical symptoms were neurological symptoms (509, 30.3%), including myalgia (311, 18.5%), headache (216, 12.8%), fatigue (83, 4.9%), and dizziness (15, 0.9%). Of these 1,682 patients, 305 patients (18.1%) admitted to the hospital were found to present with one or more digestive symptoms, including diarrhea (233, 13.9%), abdominal pain (73, 4.3%), nausea or vomiting (73, 4.3%), or poor appetite (23, 1.4%).

**Table 2:** Clinical symptoms of patients with non-critically ill COVID-19 on admission.

**Symptoms**	**No. (%)**
No. of patients	1,682
Respiratory system symptoms	1,559 (92.7)
Fever	1,177 (70.1)
Cough	951 (56.6)
Chest tightness or dyspnea	425 (25.3)
Expectoration	363 (21.4)
Chills	285 (16.9)
Sore throat	254 (15.1)
Nasal obstruction	113 (6.7)
Runny nose	107 (6.4)
Nervous system symptoms	509 (30.3)
Myalgia	311 (18.5)
Headache	216 (12.8)
Fatigue	83 (4.9)
Dizziness	15 (0.9)
Gastrointestinal symptoms	305 (18.1)
Diarrhea	233 (13.6)
Abdominal pain	73 (4.3)
Nausea or vomiting	73 (4.3)
Poor appetite	23 (1.4)

*IQR, interquartile range; COVID-19, Coronavirus disease 2019. The results were presented as number (%) for categorical variables*.

### Characteristics of Patients With Non-critically Ill COVID-19 With/Without Neurological Symptoms

Table 3 shows the characteristics of patients with/without neurological symptoms. Among these 509 COVID-19 patients with neurological symptoms, 258 patients (55.1%) were female. The median [IQR] age of patients with COVID-19 with neurological symptoms was 50 [40–58], ranging from 15 to 79 years of age. There was no significant difference in age and gender between the two groups. Compared with patients without neurological symptoms, patients with neurological symptoms had a significantly longer length of hospital stay (median [IQR], 20 (16–23) vs. 18 (14–22) days; *P* = 0.002), time of nucleic acid turning negative (median [IQR], 10 (5–14) vs. 8 (5–13) days; *P* = 0.036). Although the median [IQR] time from onset of symptom to hospital admission of the two groups is basic consistent (10 (7–15) vs. 10 (7–14) days), the mean time of patients with neurological symptoms is longer (mean ± SD [12.01 ± 6.099] vs. [10.68 ± 6.245] days; *P* = 0.001). Patients with neurological symptoms had significantly higher rates of comorbidities including hypertension (46 [9.1%] vs. 72 [6.1%]) and diabetes (29 [5.6%] vs. 24 [2.0%]). The non-critically ill COVID-19 patients with hypertension and diabetes were more likely to appear neurological symptoms.

**Table 3:** Characteristics of patients with non-critically ill COVID-19 with/without neurological symptoms.

**Characteristics**	**With neurological symptoms No. (%) (*n* = 509)**	**Without neurological symptoms No. (%) (*n* = 1,173)**	***P*-value**
Age, median [IQR], (range), years	50 [40–58], (15–79)	50 (38–58), (5–89)	0.413
Gender			0.836
Female	258 (55.1)	601 (60.0)	
Male	251 (44.9)	572 (40.0)	
From onset of symptom to hospital admission			<0.001
Mean ± SD	12.01 ± 6.099	10.68 ± 6.245	
Median [IQR] (range), days	10 (7–15), (1–30)	10 (7–14), (1–60)	
Hospital stay, median [IQR] (range), days	20 (16–23), (1–29)	18 (14–22), (1–29)	0.002
Turning to nucleic acid negative median [IQR] (range), days	10 (5–14), (0–25)	8 (5–13), (0–25)	0.036
**Comorbidities**			
Hypertension	46 (9)	72 (6.1)	0.033
Diabetes	29 (5.6)	24 (2.0)	<0.001
**Lung disease**			
Chronic bronchitis	4 (0.7)	26 (2.2)	0.042
Bronchiectasis	0	3 (0.2)	0.254
Tuberculosis	2 (0.4)	1 (0.1)	0.169
Coronary heart disease	10 (2.0)	5 (0.4)	0.794
Cerebral infarction	2 (0.4)	3 (0.2)	0.635
**Clinical symptoms**			
**Respiratory system symptoms**			
Fever	374 (73.5)	803 (68.2)	0.039
Cough	313 (61.5)	638 (54.4)	0.007
Chest tightness or dyspnea	195 (38.3)	231 (19.7)	<0.001
Expectoration	161 (31.6)	202 (17.2)	<0.001
Chills	178 (35.0)	107 (9.1)	<0.001
Sore throat	129 (25.3)	125 (10.7)	<0.001
Nasal obstruction	71 (14)	42 (3.6)	<0.001
Runny nose	63 (12.4)	44 (3.8)	<0.001
**Gastrointestinal symptoms**			
Abdominal pain	57 (11.2)	16 (1.4)	<0.001
Nausea	6 (1.2)	6 (0.5)	0.135
Vomiting	40 (7.8)	21 (1.8)	<0.001
Poor appetite	17 (3.3)	6 (0.5)	<0.001
**Clinical outcomes**			
Expansion of pulmonary infection	46 (9.0)	68 (5.8)	0.015

*IQR, interquartile range; COVID-19, Coronavirus disease 2019. P-values indicate differences between patients with and without neurological symptoms, and P < 0.05 was considered statistically significant. The results were presented as median [IQR] (range) for continuous variables and number (%) for categorical variables. The different characteristics between the two groups were tested by the Mann-Whitney U test (continuous variables) or χ^2^ test or Fisher's exact test (categorical variables)*.

Compared with patients without neurological symptoms, those with neurological symptoms presented with significantly higher rates of respiratory system symptoms, including fever (374 [73.5%] vs. 803 [68.2%]), cough (313 [61.5%] vs. 638 [54.4%]), chest tightness or dyspnea (195 [38.3%] vs. 231 [19.7%]), expectoration (161 [31.6%] vs. 202 [17.2%]), chills (178 [35.0%] vs. 107 [9.1%]), sore throat (129 [25.3%] vs. 125 [10.7%]), nasal obstruction (71 [14.0%] vs. 42 [3.6%]), and runny nose (63 [12.4%] vs. 44 [3.8%]). Patients with neurological symptoms also had significantly higher rates of gastrointestinal system symptoms including abdominal pain (57 [11.2%] vs. 16 [1.4%]), vomiting (40 [7.8%] vs. 21 [1.8%]), and poor appetite (17 [3.3%] vs. 6 [0.5%]). Additionally, we found that patients with neurological symptoms were more likely to occur expansion of pulmonary infection compared with the patients without neurological symptoms (46 [9.0%] vs. 68 [5.8%]).

## Discussion

Recent studies revealed that fever, cough, and dyspnea are the most common and typical clinical symptoms in COVID-19 patients (25, 26). However, atypical clinical manifestations including the digestive and nervous system were significant differences in different study populations (27). In this study, we found that neurological symptoms are common in patients with non-critically ill COVID-19. Among 1,682 non-critically ill COVID-19 patients, 509 patients (30.3%) had at least one neurological symptom. Compared with patients without neurological symptoms, patients with neurological symptoms had a significantly longer length of hospital stay and time of nucleic acid turning negative. Patients with neurological symptoms were more likely to occur the expansion of pulmonary infection compared with the patients without neurological symptoms (46/509 [9.0%] vs. 68/1,173 [5.8%]). These results suggest that neurological symptoms are significantly associated with the disease processes of COVID-19. Moreover, compared with patients without neurological symptoms, although the median [IQR] time of the two groups is basic consistent, the mean time from onset of symptom to hospital admission is longer in patients with neurological symptoms, it suggests that nervous system-related symptoms have not garnered much attention. Based on this finding, early identification and aggressive treatment are particularly important for COVID-19 patients with neurological symptoms.

The current study demonstrates that patients with hypertension and diabetes comorbidities are more prone to experience neurological symptoms during COVID-19. The exact pathophysiological mechanism underlying nervous system injury caused by COVID-19 is not fully understood. Genomic analysis reveals that SARS-CoV-2 belongs to betacoronavirus (βCoV) family that includes severe acute respiratory syndrome coronavirus (SARS-CoV) and Middle East respiratory syndrome coronavirus (MERS-CoV). Research has confirmed that the infection of other CoVs, such as in SARS-CoV and MERS-CoV can cause neurologic injury by invading the brain. SARS-CoV had been detected in brain tissue and cerebrospinal fluid, and some SARS patients experienced central nervous symptoms and neurological sequelae (28–30). SARS-CoV-2 shares a highly homological sequence with human SARS-CoV (with 82% identity) and infects host cells by the same angiotensin-converting enzyme 2 (ACE2) receptor (31–34). The expression of ACE2 protein was observed in multiple human organs including lung, small intestine, colon, liver, kidney, and brain; thus, these organs have been confirmed to be infected (35, 36). A recent study shows that SARS-CoV-2 leads to neuronal damage by the expression of ACE2 in central nervous system tissue (18). In addition, another study also explained that SARS-CoV-2 invades gastrointestinal tissues, and large quantities of harmful metabolites were absorbed, which causes neurological symptoms through the gut–brain axis (15). These findings indicate the expression and distribution of ACE2 in the nervous system may play a key role in the interaction between SARS-CoV-2 and nervous system injury. Hypertension or diabetes patients are always treated with ACE inhibitors and ARBs, which results in an upregulation of ACE2 expression (37, 38). We speculate that diabetes and hypertension treatment with ACE2-stimulating drugs may increase the risk of developing more neurological symptoms during COVID-19 infection.

The present study detailed investigates the neurological symptoms and implications of non-critically ill COVID-19 patients, which reminds clinicians not to overlook these neurological symptoms during treatment for COVID-19. We found 21 patients (4.1%) presented with only neurological symptoms in the absence of respiratory symptoms and digestive symptoms. In addition, recent reports suggest that anosmia or hypogeusia and dysgeusia are also one of the clinical manifestations. The current study demonstrates that neurological symptoms are significantly associated with the processes of COVID-19. Therefore, early identification and aggressive treatment are particularly important for COVID-19 patients with neurological symptoms.

This study has several limitations. First, we only collected and analyzed non-critically ill patients with COVID-19. It would be better to include more patients with severe infection. Second, we only analysis the neurological symptoms. Considering that Fangcang shelter hospitals are not regular hospitals but temporary hospitals in the special circumstances, magnetic resonance imaging, cerebrospinal fluid (CSF) test, electroencephalogram (EEG), and electromyogram (EMG) cannot be implemented. Third, this study only presents a preliminary assessment of neurological symptoms and the implications of non-critically ill COVID-19 patients. Nevertheless, the study findings should supply important information regarding the neurological implications of non-critically ill COVID-2019 patients.

In conclusion, non-critically ill COVID-19 patients commonly have neurological symptoms. Neurological symptoms reflecting nervous system injury are significantly associated with the processes of COVID-19. Early identification and aggressive treatment are particularly important for COVID-19 patients with neurological symptoms.

## Data Availability Statement

All datasets presented in this study are included in the article/supplementary material.

## Ethics Statement

The studies involving human participants were reviewed and approved by this study complied with the principles of the Declaration of Helsinki and was approved and written informed consent was waived by the ethics committee of the Zhongnan Hospital of Wuhan University (2020090K). The ethics committee waived the requirement of written informed consent for participation.

## Author Contributions

JZ had full access to all of the data in the study and took responsibility for the integrity of the data and the accuracy of the data analysis, and concept and design. NY, BM, YG, and ZX: acquisition, analysis, or interpretation of data. NY and ZX: drafting of the manuscript and statistical analysis. ZX, DL, and JZ: critical revision of the manuscript for important intellectual content. NY, ZX, and BM: administrative, technical, or material support. ZX and JZ: supervision. All authors contributed to the article and approved the submitted version.

## Conflict of Interest

The authors declare that the research was conducted in the absence of any commercial or financial relationships that could be construed as a potential conflict of interest.
